# Transgenic approaches in the pathogenic free-living amoebae: what are the hurdles?

**DOI:** 10.1017/S0031182026101772

**Published:** 2026-04

**Authors:** Jillian E. M. McKeon, Caroline M. Palmentiero, Colm P. Roster, Jaelin Ammerall, Jennifer M. Mason, James C. Morris

**Affiliations:** 1 Eukaryotic Pathogens Innovation Center, Department of Genetics and Biochemistry, Clemson University, Clemson, SC, USA; 2 Department of Genetics and Biochemistry, Clemson University, Clemson, SC, USA

**Keywords:** *Acanthamoeba* spp, *Balamuthia mandrillaris*, *Naegleria fowleri*, pathogenic free-living amoebae, transfection, transgene

## Abstract

*Naegleria fowleri, Acanthamoeba* spp., *Balamuthia mandrillaris* and *Sappinia* spp. are free-living amoebae that can infect humans and cause serious disease; therefore, these organisms are commonly referred to as pathogenic free-living amoebae (pFLA). Diagnosis and treatment of pFLA infections have historically been very challenging. If a diagnosis is made, treatment regimens currently include a combination of antifungals, antimicrobials and anticancer agents that have, to date, proven to be of little help in resolving the disease. Discovery of new therapies is critical to reduce the >90% mortality rate for the brain infections that these microbes can cause. Molecular tools that allow for the study of gene function, generation of reporter cell lines and drug target validation would greatly improve drug discovery efforts. To date, transfection approaches for use with pFLA have been limited, hindering these types of molecular studies. Based on sequence comparisons, the pFLA harbour proteins that are involved in cargo delivery to the nucleus and DNA repair mechanisms, suggesting that fundamental pathways believed to be required for stable transfection are present. However, the amoebae lack homologs to genes connected to stable maintenance of transgenes and RNA interference in other systems. While these differences may limit our ability to alter gene expression, it is also possible that unrecognized components fulfill the roles of the missing homologs. Given the value of robust transfection approaches in drug discovery and gene function studies, resolving these mechanisms would be impactful, leading to improved understanding of amoebae biology and enhancement of drug discovery efforts.

## Graphical abstract

**Figure:**
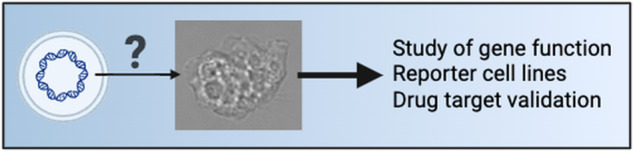

## Introduction

Pathogenic free-living amoebae (pFLA) are single-celled organisms found worldwide in soils and freshwater. The pFLA are classified as amphizoic amoebae being capable of both free-living and parasitic lifestyles (Schuster and Visvesvara, 2004). Members of this group, including *Acanthamoeba* spp., *Balamuthia mandrillaris, and Naegleria fowleri*, can cause rare, yet often fatal, central nervous system (CNS) infections in humans. A fourth amoebae, *Sappinia pedata*, has been identified as the cause of a nonlethal case of amoebic encephalitis with CNS symptoms (Gelman et al., 2001).

*N. fowleri* infection causes primary amebic meningoencephalitis (PAM), an acute, fulminant, and rapidly fatal disease. Infection occurs when amoebae-contaminated freshwater is introduced into the nasal passages. The pathogen then migrates along the olfactory nerves to the cribriform plate and gains access to the brain. Once in the brain, the amoebae feed and proliferate. They trigger host immune-mediated inflammation that, in combination with feeding amoebae, leads to tissue destruction and death of the infected individual within 7–10 days (da Rocha-azevedo et al., 2009). Current treatment of PAM involves a combination of agents, including amphotericin B, which has become a cornerstone of treatment. Other anti-infectives used in these cocktails include fluconazole, miconazole, miltefosine, azithromycin and rifampin (Grace et al., 2015). PAM is characterized by a high mortality rate (>97%) that is exacerbated by two treatment challenges. First, timely diagnosis is difficult, relying on visual identification of amoebae in cerebral spinal fluid or molecular diagnostic tests that are not widely available. Second, the efficacy of current treatments is poor, which may be partly due to delays in diagnosis preventing a timely initiation of drug treatment. While these issues are multifaceted, they highlight the need for new, rapidly acting treatments (Guemez and Garcia, 2021).

*B. mandrillaris* and several species of *Acanthamoeba* cause granulomatous amebic encephalitis (GAE), a rare opportunistic infection. Like PAM, these infections are typically fatal, with mortality rates above 90%. *B. mandrillaris* and *Acanthamoeba* spp. are found in freshwater and soil and can be introduced through skin lesions or inhalation of cysts (Krol-Turminska and Olender, 2017). GAE caused by *Acanthamoeba* mostly occurs in immunocompromised individuals, while *Balamuthia* GAE can occur in immunocompetent individuals (Kalra et al., 2020). Because of nonspecific symptoms and limited technical expertise, GAE is often misdiagnosed as bacterial or viral encephalitis (Krol-Turminska and Olender, 2017).

Current therapeutic regimens for GAE consist of a variety of drugs, including pentamidine, sulfadiazine, flucytosine, fluconazole and miltefosine. In *B. mandrillaris* GAE, a macrolide may also be included in the treatment regimen. Despite these treatments, mortality associated with the disease remains high, perhaps due to the low efficacy and high toxicity of the recommended compounds (Spottiswoode et al., 2024). Recently, nitroxoline was used to successfully treat *Balamuthia* GAE, suggesting that the agent could be a new addition to the cocktail used to treat those infections (Spottiswoode et al., 2023).

In addition to GAE, *Acanthamoeba* species can cause an infection of the cornea known as *Acanthamoeba* keratitis (AK), which can lead to vision loss (Lorenzo-Morales et al., 2015). Along with freshwater sources like pools and hot tubs, the amoebae can also proliferate in inappropriately maintained contact lens solution. AK largely impacts contact lens wearers, particularly those who do not maintain proper lens hygiene or who swim or shower wearing their lenses. Treatment approaches often drive the amoebae to differentiate into an environmentally resistant cyst stage that is refractory to many drugs. Successful management of an eye infection requires elimination of both trophozoites and cysts, a challenge that requires prolonged and repeated administration of topical antimicrobial agents (Lorenzo-Morales et al., 2015).

## Drug discovery is limited by the lack of approaches to validate potential targets

Two approaches have been used to identify drugs that are effective against amoebae. The first method uses phenotypic screens to identify agents that kill or impair amoebae growth (Rice et al., 2020). Further, these screens are typically designed to exclude agents that demonstrate unacceptable toxicity towards human cells. Promising hits are often subject to structure–activity relationship (SAR) studies to improve their drug-like properties and refine their specificity towards amoebae and away from human cell lines. Phenotypic screens have been used to identify many of the agents that constitute the current treatment cocktails (Laurie et al., 2018). The second route, target-based drug discovery, involves developing inhibitors to cellular targets anticipated to be critical for amoebae survival, including, but not limited to, structural proteins, metabolic enzymes, or DNA replication machinery (Debnath et al., 2017). Knowledge about the potential target allows biased inhibitor development using structure-guided synthesis (SGS). SGS, in tandem with SAR studies and counter screens against human homologs and cell lines, can result in highly specific inhibitors of essential amoebae cellular processes with limited toxicity against human cell lines. As an example of such an approach, we have found that inhibitors of the glucose metabolism enzyme enolase have proven to be potent anti-amebic agents with promising activity in rodent models of disease (Milanes et al., 2024).

In target-based drug development campaigns, typically one of the first steps is to confirm that the putative cellular target is critical for pathogen viability. This is usually tested using genetic approaches to ablate (by knockout) or limit expression (by RNAi or similar approach) of the target gene followed by assessment of the impact on amoebae health. If a target gene is essential, then it is likely that ablation or knockdown of the protein would be detrimental to the pathogen, justifying the pursuit of the development of inhibitors as lead compounds for drug discovery (Chatterjee-Kishore and Miller 2005; Xu et al., 2024). Additionally, genetic approaches are frequently used to confirm that observed pathogen phenotypes are due to on-target action of an inhibitor. This may involve assays that demonstrate engineered cells that no longer express a target protein, or that express a mutated target protein that no longer engages the inhibitor, lose sensitivity to the agent. The limited tools that allow for genetic manipulation of gene expression in the pFLA is a major hurdle in the validation of compounds that have shown promising activity against amoebae in culture or animal models.

## Hurdles for genetic approaches in pFLA

The ability to modify gene expression in the pFLA remains challenging, with no such tools reported for *B. mandrillaris* or *N. fowleri.* Some progress has been made in *Acanthamoeba* spp., with Peng et al. (2005) describing the transfection of cells using an ectopic vector that expressed GFP and G418 resistance from the TATA-binding protein promoter. Two other transient transfection approaches have been described that used the polyubiquitin promoter to drive expression, resulting in ∼50% transfection efficiency in each case (Hu and Henney, 1997; Kong and Pollard, 2002).

While tools for use in *N. fowleri* have not been described, approaches have been developed for the manipulation of gene expression in the non-pathogenic species *Naegleria gruberi*. For example, stable expression of GFP and antibiotic resistance has been described (Faktorova et al., 2020). In this work, the authors describe transfection of the flagellated form of the amoebae, a life form that is not found in *Acanthamoeba* spp. or *B. mandrillaris*. Transfection of *N. gruberi* trophozoites has also been reported (Nguyen et al., 2024). In that study, amoebae were transiently transfected with a modified version of the closed circular extrachromosomal ribosomal DNA element (CERE). Authentic CERE is maintained at ∼4000 copies per cell and could be a useful starting point for the development of artificial chromosomes as platforms for transgene expression.

**Figure 1.**
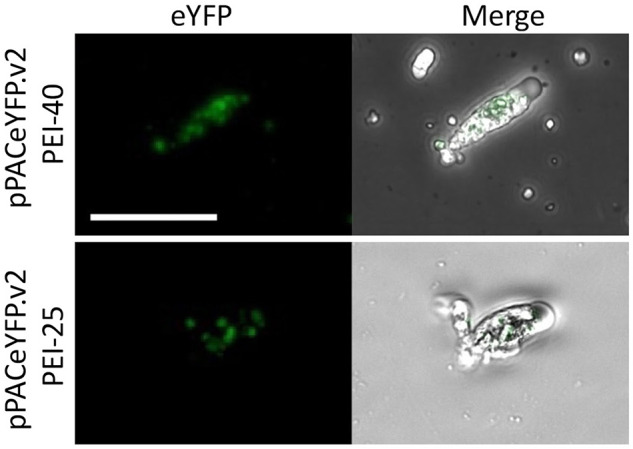
Transient transfection of *N. fowleri* (strain NfTY), with pPACeYFP.V2 yields detectable fluorescence for up to eight days before transgene expression was lost. This plasmid harbours a puromycin acetyltransferase gene (*pac)* and eYFP. Both are flanked on the 5′ side by a 1090 bp fragment amplified from immediately upstream of the *N. fowleri* ubiquitin ORF. The *pac* is flanked on the 3′ side with a 349 bp fragment of the *N. fowleri* actin UTR that is immediately downstream of the actin stop codon while eYFP is flanked by a 1000 bp of the *N. fowleri* ubiquitin gene UTR. Supercoiled plasmid (5 µg) was assembled into polyethylenimine nanoparticles and was transfected into ~2 × 10^4^ trophozoites. Briefly, the plasmid was mixed with either PEI-40 or PEI-25 at 1:1 ratio (w/w) then added to the parasites. After 48 h, cells were selected by addition of 20 µg/mL puromycin, and fluorescence was observed over time. These images were captured 8 days after transfection and 6 days after initiation of selection. The scale bar is 50 µm. Figure 1 long description.

Efforts towards transgene expression in *N. fowleri* have not progressed as far and approaches developed in *N. gruberi* have not yet been successfully adapted to the pathogen (Palmentiero, Roster, McKeon, Morris, unpublished). Gene silencing in *N. fowleri* following transfection of an episome driving dsRNA expression has been reported, but a lack of clarity over experimental details has hampered efforts to adapt these findings to general transfection approaches (Jung et al., 2008, 2009). Transient transfections, using a plasmid that drives eYFP expression from a putative ubiquitin promoter, have been successful (Figure 1), with fluorescence persisting for 8 days before detectable transgene expression is lost (Palmentiero, McKeon, Morris, unpublished).

Together, these studies highlight the limited number of tools currently available to manipulate gene expression in the pFLA. The development of a genetic toolbox to modify the pFLA is essential for the identification and characterization of novel drug targets. Here, we will address potential challenges and assess the possibility for genetic manipulation in the pFLA through interrogation of their genomes for proteins known to be involved in stable maintenance of transgenes, RNAi, and gene editing in other systems. The presence of these genes does not guarantee their role in transgenesis but may guide approach modifications required for successful gene expression manipulation in the organisms.

## Barriers to DNA delivery

The first challenge to overcome in generating transgenic amoebae is identifying methods for breaching cellular membranes to deliver cargo. One approach is not uniformly suitable for all cell types, as cellular differences can impact the efficiency of transfection approaches. For example, electroporation transfection efficiency is impacted by membrane composition. In electroporation, electric pulses are used to generate transient pores or holes in membranes that allow nucleic acids to enter the cell. Membrane fluidity, which is dependent on membrane composition, can impact pore size and pore duration.

Transfection approaches that employ cationic particles harbouring foreign DNA can also be influenced by the plasma membrane composition. Additionally, the chemical makeup and size of the lipoplexes bearing the nucleic acids can also dramatically alter transfection efficiency, further complicating optimization of approaches using cationic particles (Ramezani et al., 2009). Like the cell outer membrane, the nuclear membrane composition can impact the delivery of transfected nucleic acid to the nucleus. This membrane is selectively permeable, typically blocking large DNA plasmids from accessing the nuclear interior.

### Cellular responses to foreign DNA

Integration of foreign nucleic acid into a target cell is typically achieved using transfection or viral transduction to deliver transgenes into the cell. The fate of the nucleic acid at that point can vary, with cellular responses to nucleic acid acquired from the environment eliciting a spectrum of responses. In the following subsections, we consider the role of human proteins known to be altered in response to transfection with a focus on the potential for homologs in the pFLA.

Transfection can trigger the upregulation of mechanisms aimed at preventing viral infection or viral replication (Warga et al., 2023). The nature of the nucleic acid impacts this response, with size and single- or double-stranded nature being influential in cellular responses. For example, dsRNAs that are >30 bp are known to elicit the double-stranded RNA-dependent protein kinase (PKR) response in mammalian cells, triggering apoptosis (Lemaire et al., 2008).

At this point, it is unclear if such a mechanism, which would likely impact cell culture health, is at work in the pFLA. *N. fowleri* and *Acanthamoeba* spp. may harbour a similar response, though homologous proteins have limited identity (30% and 36%, respectively) and have been annotated as putative translation initiation factors (Table 1). RNase L, another host enzyme that can respond to viral RNA, destroys both viral and host RNA while inducing type 1 interferon signalling. Again, possible homologs in the pFLA have limited identity, raising the possibility that this activity is absent in the amoebae.

**Table 1.** pFLA orthologs of human and yeast proteins known to have changes in expression in response to transfections Table 1 long description.

Protein name	Protein ID^a^	Predicted protein amino acid number	% Identity to the human ortholog	% Identity of *Nf* to *Ac* ortholog
PKR	NfTy_047470 (3E−36)	1397	30	38
	ACA1_123230 (5E−27)	948	36	
	YMR001C (8E−25)	705	30	
	E2AK2_HUMAN (P19525)	551	100	
RNase L	NfTy_078690 (5E−16)	1375	31	28
	ACA1_129690 (1E−24)	864	32	
	YIL112W (3E−10)	1083	42	
	RN5A_HUMAN (Q05823)	741	100	
PLA2G4A	NfTy_043940 (9E − 07)	625	34	34
	ACA1_036420 (1E−04)	578	34	
	YMR008C (2E−12)	664	25	
	PA24A_HUMAN (P47712)	749	100	
PSMB8	NfTy_085550 (4E − 95)	299	60	59
	ACA1_048200 (3E−94)	275	57	
	YPR103W (1E−94)	287	54	
	PSB8_HUMAN (P28062)	276	100	
PSMB9	NfTy_049800 (2E − 72)	251	52	67
	ACA1_069610 (5E−73)	217	52	
	YJL001W (5E−59)	215	45	
	PSB9_HUMAN (P28065)	219	100	
TRIM22	NfTy_021310 (2E−07)	287	43	35
	ACA1_174460 (1E − 29)	206	35	
	YCR066W (6E−04)	487	27	
	D3DQT0_HUMAN (D3DQT0)	498	100	
ACTG2	NfTy_067520 (0)	375	85	88
	ACA1_219820 (0)	375	94	
	YFL039C (0)	375	87	
	ACTH_HUMAN (P63267)	376	100	
ACTA1	NfTy_067520 (0)	375	85	88
	ACA1_219820 (0)	375	93	
	YFL039C (0)	375	87	
	ACTS_HUMAN (P68133)	377	100	
ACTR3B	NfTy_090720 (4E−135)	422	58	58
	ACA1_396540 (0)	428	73	
	YJR065C (1E−148)	449	66	
	B7Z9W3_HUMAN (B7Z9W3)	330	100	
ACTR2	NfTy_053930 (1E−166)	389	67	71
	ACA1_074650 (0)	388	74	
	YDL029W	391	70	
	Q8IY98_HUMAN (Q8IY98)	339	100	
MYO1D	NfTy_056230 (1E−128)	1022	48	48
	ACA1_173680 (8E−130)	1135	49	
	YKL129C (2E−110)	1272	43	
	J3KRL0_HUMAN (J3KRL0)	436	100	
MYO5a	NfTy_045040 (3E−162)	1901	38	37
	ACA1_224860 (0)	2056	41	
	YOR326W (0)	1574	43	
	MY05A_HUMAN (Q9Y4I1)	1835	100	
APEX	NfTy_093310 (2E−64)	1013	46	51
	ACA1_142260 (1E−84)	328	49	
	YBL019W (4E−12)	520	25	
	APEX1_HUMAN (P27695)	318	100	

a Proteins identified with lowest *E-*value after query using human orthologs against AmoebaDB (https://amoebadb.org/amoeba/app/) or FungiDB (https://fungidb.org/fungidb/app/). *E*-values are in parentheses.

The pFLA probably lack other nucleic acid responses found in mammals, including those that are elicited by the accumulation of DNAs in the cytoplasm. DNA sensors in mammalian cells can detect cytosolic DNA and produce cyclic GMP-AMP as a second messenger that leads to type I interferon production (Sun et al., 2013), which then activates antiviral cellular responses. The gamma-interferon-inducible protein 16 (IFI16) recognizes viral dsDNA and recruits factors to induce interferon-beta expression and subsequent antiviral activity (Thompson et al., 2014). The absence of clear homologs in the pFLA to these proteins (and the interferons that are involved in the pathways) suggests the pFLA lack the canonical interferon response observed in mammals or elicit a response by distinct mechanisms.

The vehicle that is used to transfect foreign DNA can also elicit host cell responses. For example, in mammals, PLA2G4A, a phospholipase that hydrolyses phospholipids to generate the important inflammatory molecule arachidonic acid, can be activated in response to lipid introduction because of some lipofectamine transfection methods (Sarkar et al., 2020). The pFLA harbour predicted proteins with limited similarity to the human protein raising the possibility that the pFLA may harbour a similar activity. Additionally, the pFLA are noted for possessing a diverse arsenal of enzymes for the degradation of prey, so it is formally possible that one or more of these is impacting the vehicles used to deliver nucleic acids.

### Proteosome-associated proteins

Foreign proteins that can be introduced with DNA during transfection are often degraded by the immunoproteasome complex. PSMB8 and PSMB9 are key components of that complex, and *N. fowleri, Acanthamoeba* spp. and *S. cerevisiae* likely have this cellular defense mechanism given the presence of predicted homologs (Table 1; Warga et al., 2023). TRIM22, a tumour suppressor that can influence targeting of cellular proteins to the proteosome to ultimately increase reactive oxygen species (ROS), was also upregulated in mammals in response to lipofectamine-based transfection. The pFLA have predicted protein homologs with limited (43% and 35%) identity, though the polypeptides are predicted to be less than one-half the size of the human enzyme, raising the possibility of distinct cellular function.

### Delivery of cargo to the nucleus

Multiple proteins that impact transfection in mammalian cells are associated with cellular cargo movement, potentially reflecting the action of these proteins in the delivery of lipoplexes to the nucleus. These include actin and actin-associated proteins (e.g, ACTG2, ACTA1, ACTR3B and ACTR2) and myosin proteins like MYO1D and MYO5a. The pFLA amoebae have homologs of these critical proteins, but their role in transfection is unresolved (Table 1).

## DNA repair

The apurinic/apyrimidinic endonuclease (APEX), a DNA repair enzyme that removes damaged or mismatched nucleotides in base excision repair, has been implicated as having a role in mammalian transfection, including repair of abasic sites that result from transfection reagents and maintaining recipient cell genome integrity (Lopez et al., 2021). This is a conserved cellular DNA repair mechanism, so it is not surprising that the pFLA harbour putative proteins with moderate identity to the mammalian enzyme (46% and 49% for *N. fowleri* and *A. castellanii* homologs, respectively) (Table 1).

### Possible solutions to this potential barrier in the pFLA

Our understanding of the cellular repertoire of pFLA proteins that could be creating barriers to transfection through responses to foreign nucleic acids and/or vehicles used to deliver those DNAs is poor. This makes forecasting the utility of various approaches challenging and initially suggests that breakthroughs will most likely occur after rigorous testing of a breadth of transfection approaches. These, tested in combinations of conditions known to improve DNA uptake in other systems (e.g., stresses akin to serum starvation in human cells, which can improve uptake (Wallenstein et al., 2010)), will likely be required to solve this key step.

### Maintenance of foreign DNAs

Maintenance of transgenes requires parental cells to replicate and distribute the foreign nucleic acid to daughter cells. Heritable maintenance is commonly achieved through the delivery of dsDNAs as extrachromosomal episomes bearing elements required for duplication and distribution to daughter cells. Alternatively, stable transgenesis can be established through integration of the transgene into the chromosome, where it is replicated and distributed as part of the normal complement of DNA.

### Episomes and maintenance of extrachromosomal DNAs

Nuclear extrachromosomal circular DNA (eccDNA) is nearly ubiquitous amongst eukaryotes and is commonly generated through homologous recombination (HR), non-homologous end joining (NHEJ), and double-strand breaks (DSBs) (Zhao et al., 2022). Amongst the pFLA, only *N. fowleri* is known to maintain nuclear eccDNA, the closed CERE, which harbours the organism’s ribosomal RNA genes (Mullican et al., 2020). Little is known about the replication and maintenance of the CERE, with findings in the non-pathogenic species *N. gruberi* providing insight into mechanisms likely found in *N. fowleri.* In *N. gruberi*, a single origin of replication for CERE has been described from a 2.1 kb fragment of non-ribosomal DNA (Mullican et al., 2019). Additionally, in *N. gruberi* CERE is replicated through a *theta*-type replication mechanism (Mullican et al., 2019). However, the *N. fowleri* reference strain, the TY strain, has a CERE that shares limited similarity to the sequenced *N. gruberi* and *N. fowleri* CERE in the non-ribosomal sequence (43.3%) and has a high similarity to the ribosomal DNA (91.7%). The limited sequence conservation between the *N. gruberi* and *N. fowleri* CERE non-ribosomal sequences makes origin identification by sequence comparison challenging (Nguyen et al., 2021).

### Possible solutions to transgenesis using episomal DNAs in the pFLA

CERE may be a means of generating an artificial chromosome for transgene expression, like those that have been used in other systems (yeast, humans, trypanosomes (Murray and Szostak, 1983; Patnaik et al., 1996; Harrington et al., 1997)). Others have cloned the entire *N. gruberi* CERE into a bacterial vector and reintroduced the chimeric CERE into amoebae, finding that this supported maintenance of the bacterial sequence. While it is unclear if the DNA remained in the cells as episomes, the bacterial sequences could be detected by PCR after seven cell passages in the absence of selection of the transgene (Nguyen et al., 2024). To date, the origin of replication from *N. gruberi* CERE has not supported episome maintenance in *N. fowleri.* Once the origin/autonomously replicating sequence (ARS) in the *N. fowleri* CERE is resolved, plasmids bearing this sequence may be a useful means of stable introduction of foreign DNA.

## Transgene integration into the genome

Linear dsDNAs bearing transgenes can be incorporated into the genome by HR, NHEJ, or microhomology-mediated end joining (MMEJ) during chromosome replication or repair. HR requires a homologous template, usually a sister chromatid, to repair breaks, while canonical NHEJ is template-independent, requiring little to no sequence homology (<4 nt) between broken DNA ends for repair. MMEJ requires short stretches of microhomology (2–20 nt) to mediate DNA repair (Ranjha et al., 2018). Targeted gene integration likely requires HR, while NHEJ and MMEJ are sufficient for random integration, suggesting that the presence of any of these pathways could be exploited to generate transgenic amoebae by incorporation of foreign DNA into breaks during repair.

DSBs are made due to endogenous processes as well as exposure to exogenous factors. DSBs can form in unperturbed cells due to collisions between the replication and transcription machinery, nucleotide imbalances, or due to reactions with by-products of cellular metabolism (Yu and Anderson, 1997). Exposure to exogenous mutagens like ultraviolet (UV) irradiation and industrial chemicals can also damage DNA, resulting in strand breaks (Hiraku, 2025). One possibility is inducing DNA damage in amoebae which could lead to enhanced transgene integration. In support of this, introducing DSBs into the genome in other organisms including yeast and mammals results in a significant increase in random integration (Zelensky et al., 2020) as well as gene targeting events (Rudin et al., 1989; Rouet et al., 1994; Choulika et al., 1995; Smih et al., 1995).

### Proteins involved in DSB repair that may be required for transgenesis

Given the importance of DSB repair pathways in promoting integration of transgenes, it is possible that proteins involved in DNA repair could be important for the generation of stable transgenes in the pFLA. Here, we describe several of the known key players in DSB repair and consider differences found in pFLA that could explain the refractory nature of the amoebae to transgenesis.

#### The MRN complex

The MRE11-RAD50-NBS1 protein complex (the MRN complex) is one of the first repair complexes that localizes to DSBs. This complex has been proposed to have a structural role in tethering the broken chromosomes together while modulating DNA damage signalling (van den Bosch et al., 2003). The carboxy-terminal interacting protein (CtIP) has also been identified to play a critical role in promoting HR-mediated DSB repair through its interaction with the MRN complex. CtIP binds to BRCA1 as well, forming the MRN-CtIP-BRCA1 complex, which is important for end-resection to initiate HR (Wang et al., 2012).

The pFLA have a homolog for the MRE11 protein, with sequence identity ranging from 38% to 44% (Table 2). Additionally, a CtIP homolog, which may promote the MRE11 end resection of DSB, is present in all three amoebae (Ranjha et al., 2018). However, it is less clear if other protein components of the MRN complex, including RAD50 and NBS1, are present. In addition, there is no obvious BRCA1 homolog among the pFLA. The absence of BRCA homologs likely does not reflect a deficiency in homology-driven repair (HDR), as yeast lack BRCA homologs yet have very high rates of HDR (Guaragnella et al., 2014).

**Table 2.** pFLA orthologs of human and yeast HDR proteins Table 2 long description.

Protein name	Protein ID^a^	Predicted protein amino acid number	% identity to the human ortholog	% identity of *Nf* to *Ac* ortholog
MRE11	NfTy_089370 (8e−125)	680	38	41
	ACA1_064360 (1e−136)	657	44	
	YMR224C-t26_1 (3e−13)	692	41	
	MRE11_HUMAN (P49959)	708	100	
CtIP	NfTy_084080 (0 016)	883	37	0
	ACA1_167100 (6e−13)	458	43	
	YOR195W-t26_1-p1 (0 024)	821	50	
	CTIP_HUMAN (Q99708)	897	100	
RAD51	NfTy_042500 (1e − 133)^b^	913	62	70
	ACA1_071720 (0 0)	353	83	
	YER095W-t26_1-p1 (4e − 161)	400	82	
	RAD51_HUMAN (Q06609)	339	100	
RAD51B	NfTy_042500 (3e−30)	913	30	30
	ACA1_374060 (2e−44)	248	42	
	YER179W-t26_1-p1 (5e−32)	334	45	
	RA51B_HUMAN (O15315)	384	100	
RAD51C	NfTy_059920 (2e−61)	426	40	35
	ACA1_166930 (1e−87)	348	41	
	YER179W-t26_1-p1 (2e−21)	334	44	
	RA51C_HUMAN (O43502)	376	100	
RAD51D	NfTy_091520 (1e−19)	422	25	26
	ACA1_198280 (1e−19)	307	32	
	YER179W-t26_1-p1 (2e−12)	334	43	
	RA51D_HUMAN	328	100	
XRCC2	NfTy_042500 (3e−08)	913	29	62
	ACA1_201650 (8e−11)	342	28	
	YER095W-t26_1-p1 (8e−10)	400	27	
	XRCC2_HUMAN (O43543)	280	100	
XRCC3	NfTy_063090 (1e−19)	818	27	27
	ACA1_368210 (3e−42)	403	32	
	YER179W-t26_1-p1 (4e−23)	334	27	
	XRCC3_HUMAN (O43542)	346	100	
BRCA2	NfTy_059410 (1e−54)	2087	27	31
	ACA1_110350 (6e−55)	1827	30	
	YJL092W-t26_1-p1 (1 2)	1174	26	
	BRCA2_HUMAN	3418	100	
RAD54	NfTy_086900 (4e−145)	1667	47	41
	ACA1_051660 (4e−157)	689	43	
	YGL163C-t26_1-p1 (0 0)	898	48	
	RAD54_HUMAN (Q92698)	747	100	
RECQ1	NfTy_050760 (1e−147)	1405	46	41
	ACA1_378200 (3e−125)	621	42	
	YMR190C-t26_1-p1 (5e−134)	1447	41	
	RECQ1_HUMAN (P46063)	649	100	

a Proteins identified with lowest *E*-value after query using human orthologs against AmoebaDB (https://amoebadb.org/amoeba/app/) or NCBI (for yeast). *E-*values are in parentheses.

b While having greatest %identity to RAD51, this *N. fowleri* gene had the lowest *E-*value for multiple Rad51 paralogs.

#### RAD51

In mitotic cells, RAD51 is the central enzyme that carries out HR. RAD51 is an ATP-dependent DNA-binding protein that forms a nucleoprotein filament on ssDNA after end resection. The RAD51 filament searches the genome for a homologous sequence and promotes strand exchange to use the intact sequence as a template for repair (Harris et al., 2018). RAD51 interacts with several partners that promote HR in cells. For example, BRCA2 contains eight conserved ∼35 amino acid motifs called BRC repeats that can all independently interact with RAD51 to promote its nucleation on ssDNA and others stimulating the growth of the nucleofilament. RAD51 nucleoprotein filament assembly is also stimulated by RAD54 (Ranjha et al., 2018), which stimulates RAD51 DNA exchange activity. Additionally, RAD54 binds to Holliday junctions and drives branch migration while helping to stimulate DNA cleavage activity (Mazin et al., 2010).

The RAD51 paralogs, including RAD51B, RAD51C, RAD51D, XRCC2 and XRCC3, share limited sequence homology to RAD51 beyond their well-conserved ATP-binding domains. The paralogs form two major multi-protein complexes: the BCDX2 complex, consisting of RAD51B/C/D/XRCC2, and the CX3 complex, consisting of RAD51C/XRCC3. These paralog complexes have a critical role in regulating the structure and stability of RAD51 ssDNA filaments. The increased stability of the nucleofilament favours DNA repair by HR as well as increases the efficiency of the homology search. These roles are critical, as deletion of any of the paralogs inhibits the localization of RAD51 to radiation-induced DSBs and leads to a reduction in sister chromatid exchange (Harris et al., 2018).

RAD51 is an essential gene that is required for mitotic HR-mediated DSB repair. Therefore, it is not surprising that the pFLA harbour-related homologs, with *N. fowleri* and *A. castellanii* having closely related protein homologs (62% and 83% identical, respectively) (Table 2). The pFLA also harbour potential homologs of the human RAD51 paralog family (RAD51B/C/D/XRCC2/3), although modest protein identity (25–40%) emphasizes the need for experimental confirmation of these proteins. Last, the pFLA species likely harbour BRCA2 homologs, but the limited identity (27–30%) of these proteins calls for caution in assigning these functions without experimental support.

### What do similarities and differences in DNA repair suggest regarding transgenesis approaches in the pFLA?

DNA repair pathways in the pFLA may be the limiting factor in our efforts to generate transgenic pathogens. The absence of genetic tools makes understanding repair in these organisms challenging. However, numerous well-characterized inhibitors target NHEJ and HR from other organisms and could be candidates to test against the homologous targets in amoebae (Ray et al., 2020; Groelly et al., 2023). These would enable the dissection of various repair mechanisms and could prove useful for manipulating pathways that limit transgene integration (e.g., by being too high-fidelity).

## Gene editing

Gene editing, here described to alter genes in a site-directed manner through the addition, elimination, or modification of nucleotides, generates DNA breaks as part of the process. Editing occurs when NHEJ/MMEJ incorrectly repair DSBs leading to addition or deletion of nucleotides resulting in frameshift mutations. HR-mediated repair can be used to generate precise nucleotide changes (e.g., base substitutions) by providing a donor template with the desired modifications. Efforts to edit genomes in other systems have led to the development of a diverse set of reagents and approaches to generate a site-specific DSB to promote gene editing, including those that utilize transcription activator-like effector nucleases (TALENs) or CRISPR-Cas9.

TALEN proteins were originally developed from transcription activator-like effectors (TALEs) identified in the bacterial plant pathogen *Xanthomonas* (Boch et al., 2009). The TALE proteins were found to bind DNA and alter host gene transcription, much like a transcription factor. Generation of chimeric proteins bearing the DNA-binding domain of TALE with the catalytic domain of the restriction endonuclease Fok1 yielded TALENS. The Fok1 endonuclease acts as a dimer requiring two TALENS to be engineered per target site, limiting its utility for gene editing. This fusion protein introduces double-stranded DNA breaks in target genes, which use NHEJ/MMEJ or HR repair (Kim et al., 1996; Porteus and Carroll, 2005).

CRISPR-Cas9 has been developed into a powerful and versatile genome editing tool. Originally identified in bacteria, the system has been found to play an important role in bacterial adaptive immunity. Short regions of DNA separated by palindromic repeats, Clustered Regulatory Interspaced Short Palindromic Repeats (CRISPR), were found to be homologous to phages and plasmids (Bolotin et al., 2005; Mojica et al., 2005; Pourcel et al., 2005). Association of CRISPR RNA (crRNA) with Cas9, a nuclease encoded nearby the CRISPR region, yielded a target-specific ribonucleoprotein complex capable of preventing phage infection (Garneau et al., 2010; Horvath and Barrangou, 2010).

CRISPR/Cas9 cleavage specificity is directed by complementarity between the target DNA and the Cas9-associated guide RNA, allowing generation of ribonucleoprotein complexes for programmed cleavage of specific DNA sites (Jinek et al., 2012; Ran et al., 2013). The system has been simplified through an improved understanding of the components required for high fidelity editing, now only requiring the Cas9 nuclease and a single guide RNA that serves as a scaffold for interacting with the nuclease while also providing sequence specificity for the cleavage. This has made the approach a flexible and user-friendly tool for gene editing (Gaj et al., 2016).

### What are the challenges and solutions to using gene editing in the pFLA?

Gene editing will likely prove to be a useful tool for developing transgenic pFLA, as CRISPR/Cas9 has been found useful for gene manipulation in many eukaryotic systems. Direct delivery of exogenous recombinant purified Cas9 complexed with *in vitro* synthesized guide RNAs or transfection of plasmids harbouring genes for the enzyme and guide RNA may be a potential means of delivery of the activity into cells. Donor repair DNA templates could be included or delivered independently. In either case, cargo delivery approaches will need to be developed and refined first before this tool can be thoroughly assessed.

## RNAi and the pFLA

RNA interference (RNAi) is another useful tool for the manipulation of gene expression in many systems. RNAi is triggered by introducing dsRNA into cells, leading to the degradation of cognate mRNAs bearing the same sequence as the dsRNA (Fire et al., 1998). DsRNAs are processed to ∼20 bp small interfering RNAs (siRNAs) that are associated with the RNA-induced silencing complex (RISC), leading to homology-dependent degradation of the target mRNA during RNAi (Hamilton and Baulcombe, 1999; Piatek and Werner, 2014). RNAi has advantages over other approaches, including offering the opportunity to study the impact of the loss of essential genes on cells. Unlike knockouts, RNAi attenuates gene expression through reduction of mRNA and the rate of lethality is impacted by the penetrance of the silencing and the half-life of the mRNA. This makes RNAi a powerful approach for exploring gene function on a genome-wide scale (Hannon, 2002; Morris et al., 2002; Wilson et al., 2013).

### Key proteins involved in the RNAi pathway

#### Dicer

Dicer is the RNase III responsible for cleavage of dsRNAs into siRNAs, activating the RISC, which occurs in the first stages of RNAi. It is unclear if the pFLA harbour homologs of this protein as predicted proteins with the best *E*-values (thereby being unlikely to be chance events but rather reflective of a potentially shared ancestry) have low identity (24% and 26%, for *N. fowleri* and *A. castellanii* proteins, respectively) and differ from the human enzyme in size; however, both have a predicted RNase III catalytic domain, which is similar to that found in Dicer (Table 3). Adding to the complexity of this comparison, humans harbor a related RNase III, Drosha, which cleaves endogenous RNAs that have hairpin structures into microRNAs (miRNAs) that regulate gene expression. The same *A. castellanii* predicted protein had the best *E*-value for both Dicer and Drosha, raising the possibility of a function in either (or both) pathways.

**Table 3.** pFLA orthologs of human RNAi proteins Table 3 long description.

Protein name	Protein ID^a^	Predicted protein amino acid number	% identity to human ortholog	% identity of *Nf* to *Ac* ortholog
Dicer	NfTy_057690 (2E−29)	1524	24	21
	ACA1_291000 (5E−12)^b^	805	26	
	DICER_HUMAN /NP_001382612.1	1922	100	
Drosha	NfTy_028160 (8E−21)	548	29	29
	ACA1_291000 (2E−78)^b^	805	40	
	RNC_HUMAN/NP_001369437.1	1374	100	
Argonaute RISC component 1	NfTy_031470 (1E−53)^c^	1079	26	28
	ACA1_071510 (2E−18)^c^	713	22	
	AGO1_HUMAN/KAI4079798.1	857	100	
Argonaute RISC catalytic component 2	NfTy_031470 (4E−52)	1079	26	28
	ACA1_071510 (3E−18)	713	23	
	AGO2_HUMAN/KAI4012086.1	859	100	
Argonaute RISC catalytic component 3	NfTy_031470 (2E−48)	1079	25	28
	ACA1_071510 (3E−18)	713	29	
	AGO3_HUMAN/KAI4079802.1	860	100	
Argonaute RISC component 4	NfTy_031470 (6E−45)	1079	23	28
	ACA1_071510 (3E−17)	713	22	
	AGO4_HUMAN/KAI4079795.1	861	100	
Mov10 RISC complex RNA helicase	NfTy_005530 (7E−45)	1044	28	62
	ACA1_077250 (1E − 46)	1142	29	
	MOV10_HUMAN/KAI4081993.1	1003	100	
C3PO	NfTy_059820 (3E−45)	250	36	31
	ACA1_107590 (2E−45)	252	39	
	TSN_HUMAN/Q15631.1	228	100	

a Proteins identified with lowest *E*-value after query using human orthologs against AmoebaDB (https://amoebadb.org/amoeba/app/) or NCBI (for human). *E-*values are in parentheses.

b ACA1_291000 was also the only potential homolog identified for Dicer in *A. castellanii*, with a *p*-value of 5E-12 and 26% identity to the human protein.

c While humans have four argonaute RISC components, the pFLA have a single predicted ortholog.

#### Argonaute

In humans, Argonaute is composed of four related proteins. These proteins are a component of the RISC, serving to cleave the target mRNA strand that is complementary to the siRNA. The pFLA harbour single potential homologs to the four proteins (based on *E*-value, with *N. fowleri* having the best score), though the identity to the human proteins is low (Table 3).

#### MOV10

MOV10 is an RNA helicase component of the RISC that likely plays a role in resolving secondary structures that can be components of RNA. This helicase has additional activities in development and in antiviral responses (Nawaz et al., 2022). *N. fowleri* and *A. castellanii* harbour potential homologs that share 62% identity with each other.

#### C3PO

The RNAi regulator C3PO is a complex of a Mg^2+^-dependent endoribonuclease that removes the siRNA passenger strand cleavage products, which promotes the action of the RNAi machinery (Liu et al., 2009). Both *N. fowleri* and *A. castellanii* harbour possible homologs that are similarly sized to the human enzyme, although identity of the proteins was modest (36% and 39%).

Notably, homologs of several key putative RNAi proteins were not identified in the pFLA genomes. For example, DGCR8 (DiGeorge syndrome critical region gene 8, also called Pasha), a microprocessor complex subunit and binding partner of Drosha that participates in the repair of UV photoproducts, was not identified (Calses et al., 2017). While this could reflect the absence of miRNA pathways in the pFLA, the identification of conserved miRNAs in *A. castellanii* suggests the amoebae may use mechanisms distinct from other organisms to generate regulatory small RNAs (Edelbroek et al., 2024).

The pFLA also lack clear homologs of the RISC loading complex transactivation response RNA-binding protein, TRBP2, and the protein kinase RNA activator (PACT). Both proteins are part of the dsRNA-binding components of RISC, with TRBP2 also participating in the loading of AGO2 to the complex while PACT functions to regulate processing of pre-siRNA substrates. Both proteins also participate in the PKR.

### Outlook for using RNAi in the pFLA

RNAi is not a universal eukaryotic activity, with some organisms lacking the ability to silence gene expression. These include the model species *Saccharomyces cerevisiae*, which lacks key proteins like Dicer and Argonaute. The pFLA have homologs to those proteins, suggesting they may have an intact RNAi pathway. The lack of clear understanding on optimized delivery of the dsRNA triggers required for RNAi (by transfection or similar) creates challenges in exploiting the pathway for manipulating gene expression. The giant virus *Catovirus naegleriensis* may provide clues on how to transfect the amoebae. This virus can infect and kill bacteria-fed *Naegleria* but does not infect amoebae adapted to axenic conditions. This suggests that the virus requires phagocytosis for entry into the host – a path we have attempted to exploit by feeding *N. fowleri* bacteria that express dsRNAs without success (McKeon and Morris, unpublished).

## Additional considerations

This review has focused on cellular responses to transfection that could be impacting nucleic acid uptake. There are additional hurdles that need to be addressed before routine transfection approaches are resolved. First, it is unclear what nucleic acid elements are required for transgene expression. Regulatory sequences like promoters will likely be key components of a transgene expression system. These regulatory sequences, in combination with useful selectable markers, will also be important for development of heritable transgene expression tools. Other regulatory components, including polyA addition sequences and terminators, may be required for this approach to work.

## Conclusions

The pFLA are responsible for infections that are difficult to treat and typically have poor outcomes. Efforts to develop new therapeutics for the treatment of these infections have been hampered by a lack of understanding of the basic cell biology of the organisms. As a result, amoebae-specific treatment options are not available. Instead, repurposed agents have been identified that can be anti-amebic *in vitro*, but these have yet to be proven effective in the treatment of disease.

Resolving approaches for genetic manipulation of the pathogens would open the door to new treatment avenues providing a clearer understanding of amoebae gene function. This would have a 2-fold impact, enabling the selection of targets that are amoebae-specific and the assessment of their essentiality to the organism. Unfortunately, the challenges associated with transfection of the pFLA have limited the use of reverse and forward genetics to study gene function, validate drug target choices and create reporter cell lines.

The reasons for these challenges are likely multifaceted. First, the developmental stage of the amoebae may influence transfection efficiency. For example, transfection of *N. gruberi* flagellates has been reported (Faktorova et al., 2020), although this approach has not been successfully adopted to *N. fowleri* (Palmentiero and Morris, unpublished observation). The cell cycle stage may also be critical for success, although this has not yet been explored in *N. fowleri*. In *N. gruberi*, CERE abundance increases during encystment, possibly in preparation for future excystment when the environment is more nutrient-rich (Nguyen, 2024). This increase in copy number may impact transfection efficiency of exogenous DNA by depleting biosynthetic intermediates needed for the replication and repair of the transgenic episome. Second, successful transgenesis may require cells to be actively phagocytosing food/prey, rather than using pinocytosis associated with axenic growth to acquire nutrients. Supporting this supposition, viral uptake is enhanced in phagocytosing amoeba.

Other potential inhibitors of transfection include cellular nucleases. In humans, DNase II, an acidic lysosomal endonuclease, can impact transfection efficiency of foreign DNAs (Howell et al., 2003). While the amoebae lack a clear homolog to this enzyme (by sequence comparison), other endonucleases may be at work impacting transfection, including those used to break down prey nucleic acids. Alternatively, these could include the intron-encoded homing endonucleases (Elde et al., 1999, 2000), though their restrictive sequence specificity suggests this is unlikely.

This review explores various cellular pathways known to influence transfection in a variety of systems, using sequence gazing to assess the likelihood of the presence of homologs in the pFLA. The lack of sequence homologs for various cellular processes potentially related to transgenesis cannot rule out the possibility that other unrecognized proteins provide the activity. To fully validate potential candidates, heterologous expression in tractable systems, such as yeast, followed by careful assessment of function, could be used. Nevertheless, multiple missing conserved components raise the possibility that a pathway may be diminished or absent in the pathogens.

## Figure 1 Long description

A two-by-two layout with two rows and two columns. The left column is labeled “eYFP” at the top and the right column is labeled “Merge” at the top. Along the left side, the top row is labeled “pPAccEYPv2” on one line and “PE-404” on the next line; the bottom row is labeled “pPAccEYPv2” on one line and “PE-152” on the next line. Top-left: Black background with multiple small green puncta clustered near the center-left. A white horizontal scale bar is located near the lower-left. Top-right: Grayscale background with a bright, elongated oval object near the center. Green puncta overlay the object. Several small bright circular spots are scattered around. Bottom-left: Black background with a small cluster of green puncta near the center. Bottom-right: Grayscale background with an irregular bright object near the center. Green puncta overlay the object. A few small bright circular spots are scattered around.

Navigate back to Figure 1.

## Table 1 Long description

The table lists predicted orthologs for several human proteins, giving each organism’s protein ID, predicted amino acid length, percent identity to the human ortholog, and for Nf the percent identity to the Ac ortholog. For each protein group, entries are provided for Nf, Ac, yeast, and the human reference. Actin-related proteins are the most conserved: ACTG2 and ACTA1 show 85 percent identity for Nf to human and 94 or 93 percent for Ac to human, with Nf to Ac identity at 88 percent; ACTR2 and ACTR3B are also relatively high at 67 and 58 percent for Nf to human. Proteasome subunits are moderately conserved, with PSMB8 at 60 percent and PSMB9 at 52 percent identity for Nf to human, and Nf to Ac identity of 59 and 67 percent. Innate immune-related proteins show lower conservation: PKR is 30 percent identity for Nf to human and RNase L is 31 percent, with Nf to Ac identity of 38 and 28 percent. Several amoeba proteins are much longer than the human references, such as PKR and RNase L in Nf, indicating that percent identity reflects sequence similarity rather than matching full-length proteins. Orthologs were selected based on the lowest database search E-value, so results depend on database content and may not capture all functional differences.

Navigate back to Table 1.

## Table 2 Long description

The table lists predicted orthologs for multiple homologous recombination and DNA damage response proteins across Naegleria fowleri, Acanthamoeba castellanii, yeast, and human. For each protein group it reports the candidate protein identifier with a database search E-value, predicted amino acid length, percent identity to the human protein, and for N. fowleri the percent identity to the Acanthamoeba ortholog when available. In N. fowleri, identities to human range from about 25 to 62 percent, with RAD51 highest at 62 percent and RAD54 and RECQ1 also relatively high at 47 and 46 percent. RAD51 paralogs in N. fowleri are lower: RAD51B 30 percent, RAD51C 40 percent, RAD51D 25 percent, XRCC2 29 percent, and XRCC3 27 percent. N. fowleri similarity to Acanthamoeba is often in the mid 20s to low 40s, but RAD51 is higher at 70 percent and XRCC2 is 62 percent. Protein lengths vary widely, from a few hundred amino acids for many RAD51 family members to over two thousand for BRCA2 in N. fowleri. Some N. fowleri entries reuse the same gene identifier across several RAD51 paralog rows, indicating the best-hit gene may match multiple related paralogs and should be interpreted cautiously.

Navigate back to Table 2.

## Table 3 Long description

The table lists predicted orthologs of human RNA interference proteins in two pFLA species, giving each candidate protein ID with its search significance, predicted protein length, percent identity to the human ortholog, and percent identity between the two amoeba orthologs. For Dicer, NfTy_057690 is 1524 amino acids and 24 percent identical to human, while the A. castellanii candidate ACA1_291000 is 805 amino acids and 26 percent identical to human; the Nf to Ac identity for Dicer is 21 percent. For Drosha, NfTy_028160 is 548 amino acids and 29 percent identical to human, and ACA1_291000 is 805 amino acids and 40 percent identical to human; Nf to Ac identity is 29 percent. All four human Argonaute entries map to the same Nf candidate, NfTy_031470 at 1079 amino acids, with human identities ranging from 23 to 26 percent and a consistent Nf to Ac identity of 28 percent; the A. castellanii ortholog ACA1_071510 is 713 amino acids with human identities from 22 to 29 percent depending on the Argonaute. Mov10 shows 28 to 29 percent identity to human, but the highest cross species similarity in the table, with 62 percent identity between NfTy_005530 and ACA1_077250. C3PO shows the highest identity to human among the listed proteins, at 36 percent for NfTy_059820 and 39 percent for ACA1_107590, with 31 percent identity between the amoeba proteins. Human reference proteins are included as 100 percent identity baselines, and the reported matches depend on database searches and best hit selection, so they indicate predicted homology rather than confirmed function.

Navigate back to Table 3.
